# Supplementary material for: Complex regulatory networks influence pluripotent cell state transitions in human iPSCs
Source: Nat Commun. 2024 Feb 23;15:1664. doi: 10.1038/s41467-024-45506-6 (PMC10891157; doi:10.1038/s41467-024-45506-6)
Supplement: Supplementary file 1 — Supplemental Information [file 41467_2024_45506_MOESM1_ESM.pdf]

SUPPLEMENTAL DATA LEGENDS

Supplemental Data 1. iPSCORE Subject Information.

Information about each of the 219 iPSCORE individuals that were included in this study, including subject iPSCORE ID (Column A), subject universally unique identifier (UUID, Column B), whole genome sequencing sample UUID (Column C), sex (Column D), age at enrollment (Column E), if there is corresponding RNA-seq data (Column F), ATAC-seq data (Column G), and the top 20 genotype PCs for global ancestry (Columns H-AA) for the individual. The kinship matrix describing the relatedness of the samples was deposited on Figshare.

Supplemental Data 2: RNA-seq Sample Information

Information about the 213 RNA-seq samples used in this study, including subject iPSCORE ID (Column A), subject universally unique identifier (UUID, Column B), RNA-seq sample UUID (Column C), the iPSC iPSCORE ID which consists of the iPSCORE ID, iPSC clone number and the iPSC passage number (Column D), the iPSC clone (Column E) and passage (Column F) numbers, the number of properly paired reads (Column G), the estimated formative proportion (Column H), and the CIBERSORT deconvolution correlation values (Column I).

Supplemental Data 3: Signature Gene Matrix for RNA-seq Cellular Deconvolution

Signature gene expression matrix for CIBERSORT cellular deconvolution of 213 RNA-seq samples. The table includes the Gencode v34 gene ID and gene name (Columns A and B), the expression (TPM) of 300 differentially expressed genes between the FACS-sorted formative (GCTM-2^{high}CD9^{high}EPCAM^{high}, Column C) and primed (unsorted, column D) populations from Lau et al. 2020[1].

Supplemental Data 4: Gene Network Module Memberships

Information about gene network module (GNM) analysis annotations for the 16,110 expressed genes, including the Gencode v34 gene ID (Column A) and gene name (Column B), the corresponding GNM (Column C), whether the corresponding GNM was used for downstream analyses (Major GNM, Column D), whether the gene was considered Pareto (Column E), the gene's co-expression degree connectivity across all 16,110 expressed genes (genome-wide degree, Column F), and the gene's co-expression degree connectivity (Column G) within its corresponding module (intramodular degree).

Supplemental Data 5: GNM Enrichment Results

Results from the Fisher's Exact test to calculate pluripotency cell state gene set enrichments. Information includes the annotation tested (Column A), the GNM tested (Column B), the odds ratio (Column C), the two-sided p-value (Column D), and the Benjamini-Hochberg corrected p-value (Column E).

Supplemental Data 6: ATAC-seq Sample Information

Information about the 263 individual ATAC-seq samples from iPSCORE individuals that were merged by library into 150 ATAC-seq samples. The table includes the subject universally unique identifier (UUID, Column A), the iPSCORE subject ID (Column B), the iPSC iPSCORE ID which consists of the iPSCORE subject ID, clone number and passage number (Column C), sample UUIDs for the 150 merged ATAC-seq libraries (Column D), sample UUIDs for the 263 ATAC-seq samples before merging (Column E), the iPSC clone number (Column F), the iPSC passage (Column G) and number of reads passing filters (Column H), the mean fragment size (Column I), the number of broad peaks used for QC (Column J), the ratio of 100bp reads to 150bp reads in the merged sample (1=100% 100bp reads and 0=100% 150bp reads; see Methods; Column K), the estimated formative proportion (Column L), the CIBERSORT deconvolution correlation values (Column M), and whether the merged sample was used as a reference for establishing a set of reference narrow peaks (Column N). **Note:** To obtain non-redundant data for the 150 merged ATAC-seq samples used in downstream analyses, use the unique rows from columns A-D and F-M.

Supplemental Data 7: Signature Peak Matrix for ATAC-seq Cellular Deconvolution

Signature gene expression matrix for CIBERSORT cellular deconvolution of 150 ATAC-seq samples. The table includes the narrow peak ID (Column A), the accessibility (TMM) of 200 differentially accessible peaks between the FACS-sorted formative (GCTM-2^{high}CD9^{high}EPCAM^{high}, Column B) and primed (GCTM-2^{mid}-CD9^{mid}, column C) populations from Lau et al. 2020.

Supplemental Data 8: Regulatory Network Module (RNM) Memberships and Annotations

Information about regulatory network module (RNM) analysis annotations for the 56,978 accessible peaks, including the peak ID (Column A), peak chromosome, start and end positions (Column B-D), the corresponding RNM (Column E), whether the RNM was one of the 13 used for downstream analyses (Major RNM, Column F), whether the peak was considered a Pareto peak (Column G), the peak's co-accessibility degree connectivity (Column H) across all 56,978 accessible peaks (genome-wide degree), and the peak's co-accessibility degree connectivity (Column I) within its corresponding module (intramodular degree), the iPSC-18 ChromHMM chromatin state annotation (Column J), the collapsed chromatin states (Column K), and the Gencode v34 gene ID (Column L), gene name (Column M), the

distance in base pairs (Column N) of the closest expressed gene after ROCK kinase inhibitor stimulation (see Methods), and whether the peak overlaps a Formative (Column O) or Primed (Column P) peak.

Supplemental Data 9: TOBIAS Predicted Binding Site Validation

This table includes information about the TOBIAS prediction validation analysis, including the ENCODE ID of the TF ChIP-seq data for H1 ESCs (Column A), the corresponding transcription factor (Column B), the two-sided p-value and the odds ratio for the Fisher's Exact tests (Columns C-D).

Supplemental Data 10: Transcription Factor Group Motif Memberships

Information about TF groups determined by TOBIAS predicted binding similarities for 187 motifs, including the HOCOMOCO motif ID (Column A), the Gencode v34 gene ID (Column B), gene name (Column C) and the name of the collapsed TF group to which the motif belongs (Column D). **Note:** The TOBIAS motif distance matrix and predicted binding sites for all 187 motifs were uploaded to GEO (GSE203377) and FigShare (136585).

Supplemental Data 11: Annotations of 56,978 peaks for binding of 92 TF groups

This table includes the TOBIAS-predicted transcription factor binding sites for all 56,978 ATAC-seq peaks. Information includes; the peak ID (Column A), and binary annotations for the 92 collapsed TF groups and "Not Bound" peaks (Columns B-CP), where 1 indicates that there is a bound TF group on the corresponding peak.

Supplemental Data 12: RNM Annotation Enrichment Results

This table contains the Fisher's Exact test results for enrichments in the RNM Pareto peaks, including the annotation type (transcription factor, chromatin state, and cell state, Column A), the corresponding annotation (Column B), the tested RNM (Column C), the odds ratio, two-tailed p-value, and Benjamini-Hochberg corrected p-value (Columns D-F).

Supplemental Data 13: RNM Fetal Tissue ATAC-seq Enrichment Results

This table contains the Fisher's Exact test results for the single-cell fetal tissue-specific peak RNM enrichments, including the tested fetal cell type (Column A), the tested RNM (Column B), the odds ratio, two-tailed p-value, and Benjamini-Hochberg corrected p-value (Columns C-E).

Supplemental Data 14: Fetal Tissue TFBS Enrichment Results

This table contains the Fisher's Exact test results for the single-cell fetal tissue-specific peak TFBS enrichments, including the tested fetal cell type (Column A), the tested TFBS (Column B), the odds ratio, two-tailed p-value, and Benjamini-Hochberg corrected p-value (Columns C-E).

Supplemental Data 15: Allele-Specific Chromatin Accessibility (ASCA) Results

This table contains information on SNPs tested for ASCA, including SNP ID and gnomad RSID (Columns A-B), Peak ID (Column C), number of reads mapping to the reference and alternative alleles (Columns D-E), the allelic imbalance fraction (Column F), the number of heterozygous individuals tested (Column G), the minor allele frequency (Column H), the two-sided p-value from the binomial test and Benjamini-Hochberg corrected p-value (Columns I-J), whether the SNP has ASCA (adjusted P-value < 0.05, Column K).

SUPPLEMENTAL NOTE 1

Weighted gene co-expression network analysis (WGCNA)[6] is the most commonly used gene module detection method, however, it cannot account for kinship (genetically related individuals). In this study, we used hiPSC lines from 219 individuals (Supplemental Data 1) recruited as part of the iPSCORE resource, of which 140 belonged to families composed of two or more subjects (range: 2–14 subjects). To address this confounding factor, we first applied an LMM to calculate gene co-expression and ATAC-seq peak co-accessibility, using kinship as the random effects term (See Methods). We loaded the edges of the significantly co-expressed genes and co-accessibility ATAC-seq peaks into a network and applied the Leiden community detection algorithm to detect modules. To determine if our approach or WGCNA is more suitable for module detection using iPSCORE resource samples**,** we applied WGCNA to calculate gene co-expression in the 213 hiPSC RNA-seq samples. Downstream analyses showed that the WGCNA modules were correlated with biological and technical covariates, as well as family structure (Supplemental Figure 3). We also determined that the LMM-Leiden module detection approach was more precise at identifying modules associated with formative-state-specific gene expression than WGCNA (Supplemental Figure 4). These results show that the conventional WGCNA module detection approach can be affected by donor relatedness and that accounting for kinship leads to more accurate module membership.

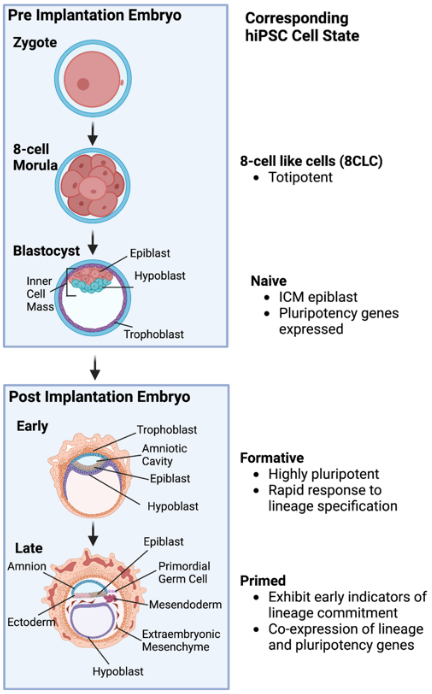

Supplemental Figure 1. Pluripotency cell states in human induced pluripotent stem cell lines.

Recent studies have shown that human induced pluripotent stem cell (hiPSC) lines are mosaics, composed of varying proportions of cells in different, interconvertible pluripotent cell states with distinct transcriptional circuitry and epigenomic profiles[1]. Live cell staining[1] and single-cell analyses[2] in hiPSCs have revealed that the interconvertible pluripotent cell states have morphological, transcriptional, and epigenetic profiles that correspond to different embryonic and extraembryonic lineage fates[1,2]. Subpopulation heterogeneity complicates the molecular characterization of these interconvertible stem cell stages. Most cells within a conventional hiPSC culture resemble the late post-implantation epiblast stem cells[1], which resembles the primitive streak and are referred to as “primed”. Primed stem cells co-express lineage-specific and pluripotency genes. “Naïve” stem cells represent the cellular state of the inner cellular mass (ICM) preimplantation epiblast which gives rise to the embryo proper. At this same pre-implantation stage are the extra-embryonic primitive endoderm cells (PrE) that give rise to the primary yok sac. A pluripotent state called “formative” has been identified as developmentally between the naïve and primed states, which represents the early post-implantation epiblast (EPE). The formative pluripotent stage has been shown to be comprised of cells enriched for high self-renewal[1]. Typically, a small proportion of cells within a hiPSC line are totipotent and resemble the 8-cell morula (8 cell-like cells; 8CLC)[3]. The 8CLC subpopulation is enriched in catabolic processes, such as protein synthesis and RNA metabolism[3]. Created with BioRender.com**.**

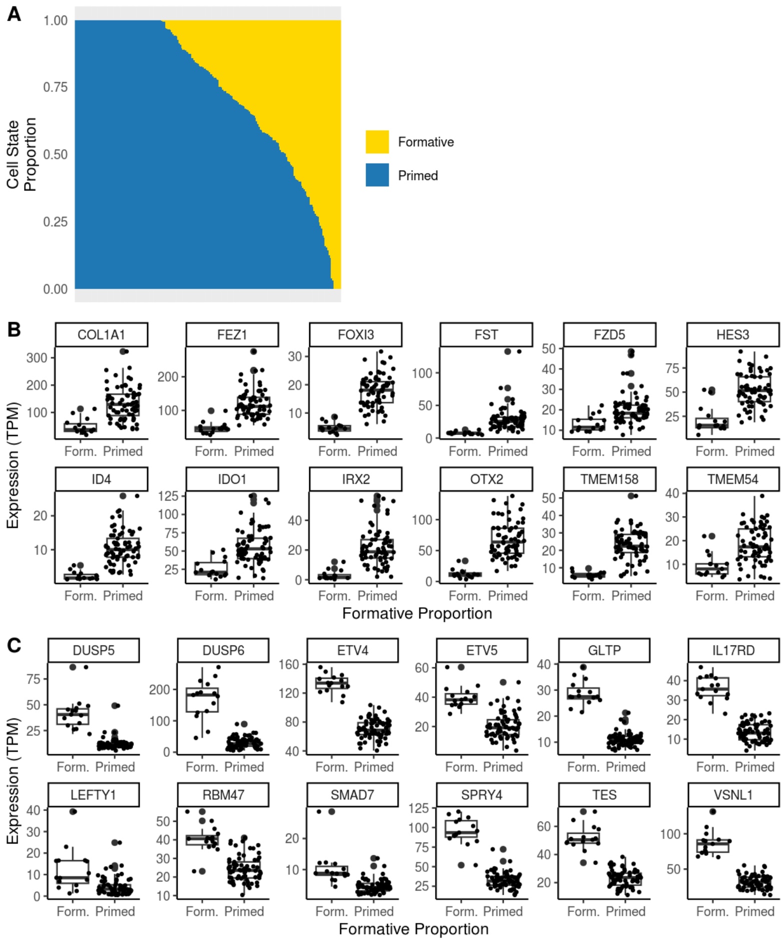

Supplemental Figure 2. Formative cell state deconvolution of 213 hiPSC lines

(A) Stacked bar plot showing estimated proportions of cells in formative and primed states across 213 hiPSC lines. Each bar on the x-axis represents a hiPSC line with the corresponding estimated proportions of each cell state on the y-axis. We generated gene signatures using bulk RNA-seq data for FACS-sorted formative and paired unsorted (e.g. primed) cells[1]. While there are multiple pluripotency states present in hiPSCs, the analysis was limited to estimating the fraction of cells that were formative-like (e.g. formative, naïve, totipotent) and the fraction that were primed-like (e.g. general population). We applied the CIBERSORT deconvolution algorithm with a signature matrix containing the 300 most differentially expressed genes between the two populations (Supplemental Data 2), and observed that the estimated fraction of cells in the formative state exhibited a wide range (0-100%) across the 213 hiPSC lines. Of note, while the RNA-seq and ATAC-seq data were generated from the same hiPSC lines, there were batch effects because of differences in culture conditions (i.e., the ATAC-seq data were generated from hiPSCs that had been cultured with ROCK inhibitor; see Methods). The estimated ratios of formative:primed cells differ between the deconvolution analysis using RNA-seq data and the deconvolution analysis using

ATAC-seq data shown in Figure 2E. We feel that technical differences (the aforementioned batch effects and the FACs sorted cell populations used to generate the gene and peak signatures were slightly different[1]) underlie most of the variance in the estimated ratios of formative:primed cells but biological factors may also contribute.

(B) Boxplots showing the differential expression of 12 primed-specific signature genes between ten hiPSC lines with the lowest and highest estimated formative proportion. The boxplot maxima and minima are set by the samples with the highest and lowest expression of the corresponding gene, the center line represents the median gene expression, the upper and lower hinges correspond to the 25th and 75th percentiles, and the upper and lower whiskers extend to the highest and lowest value within 1.5 times the interquartile range (IQR).

(C) Boxplots showing the differential expression of 12 formative-specific signature genes between ten hiPSC lines with the lowest and highest estimated formative proportion. The boxplot maxima and minima are set by the samples with the highest and lowest expression of the corresponding gene, the center line represents the median gene expression, the upper and lower hinges correspond to the 25th and 75th percentiles, and the upper and lower whiskers extend to the highest and lowest value within 1.5 times the interquartile range (IQR).

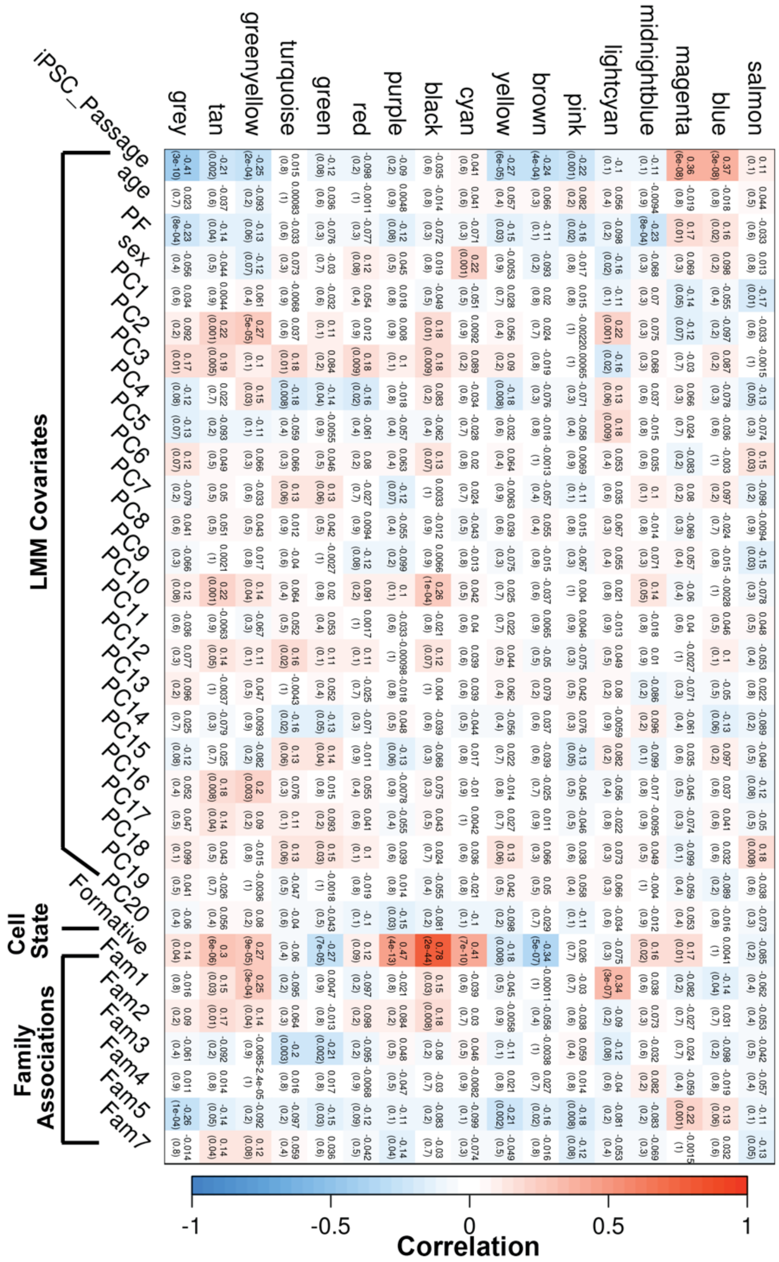

Supplemental Figure 3. Heatmap demonstrating genetic factors influence WGCNA gene module detection

WGCNA[6] is the most commonly used R package for gene module identification. However, WGCNA cannot account for data that includes samples from related individuals or correct for covariates. To evaluate whether WGCNA gene module identification was affected by the presence of related individuals, we applied it to the 213 hiPSCs RNA-seq data using the standard workflow outlined in the tutorial and identified 17 modules (top referred to by colors). We used the WGCNA *corPvalueStudent* function to calculate associations between the 17 WGCNA gene modules and the covariates used in our LMM (age,

sex, hiPSC passage, number of reads passing filters, and 20 ancestry PCs), as well as estimated formative (EPE) proportion, and binary annotations for large families (Fam 1, 2, 3, 4, 5 and 7). Each cell is filled with correlation (top value) and the p-value of association (bottom value). Several covariates exhibited significant associations with several modules. For example, the "black" module is significantly associated with global genotype PCs 2, 3, 10, and samples from families 1 and 2, and the formative-state cell proportion. In total, 11 modules were significantly associated with global genotype PCs, and 13 modules were associated with specific families. This indicates that genetic factors influence WGCNA detection of biologically relevant modules.

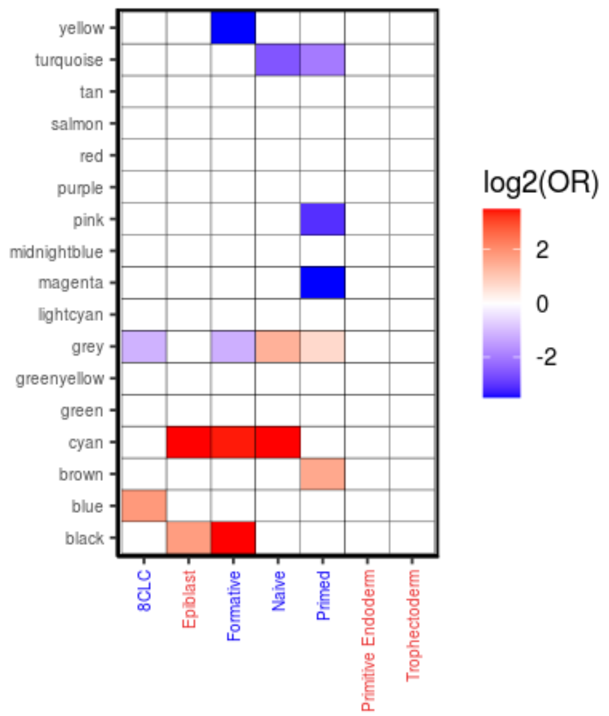

Supplemental Figure 4. Pluripotent cell state enrichments in WGCNA gene modules

There is no consensus on gene module validation[6] , however, assessing the biological enrichments is commonly used to determine which modules capture genes involved in the same biological processes. We demonstrated that our LMM approach using kinship as a random effects term to identify significantly co-expressed genes, followed by loading the edges of the co-expressed genes into a network and applying the Leiden community detection algorithm to detect modules identified a single GNM (5) enriched with formative-specific genes (Figure 1J-K). To evaluate WGCNA precision, we re-performed the stem cell state enrichment analysis (Figure 1J) using the WGCNA modules (Supplemental Note 1). Published gene set labels on the y-axis were colored to indicate whether they were curated from *in vivo* (red) or *in vitro* (blue) experiments (Supplemental Data 5). We observed that the formative-state gene set is strongly enriched in the "black" and "cyan" WGCNA modules (Supplemental Figure 3). This suggests that in the iPSCORE cohort, which includes samples from related individuals, our LMM model more precisely identifies gene module(s) associated with the formative state than WGCNA.

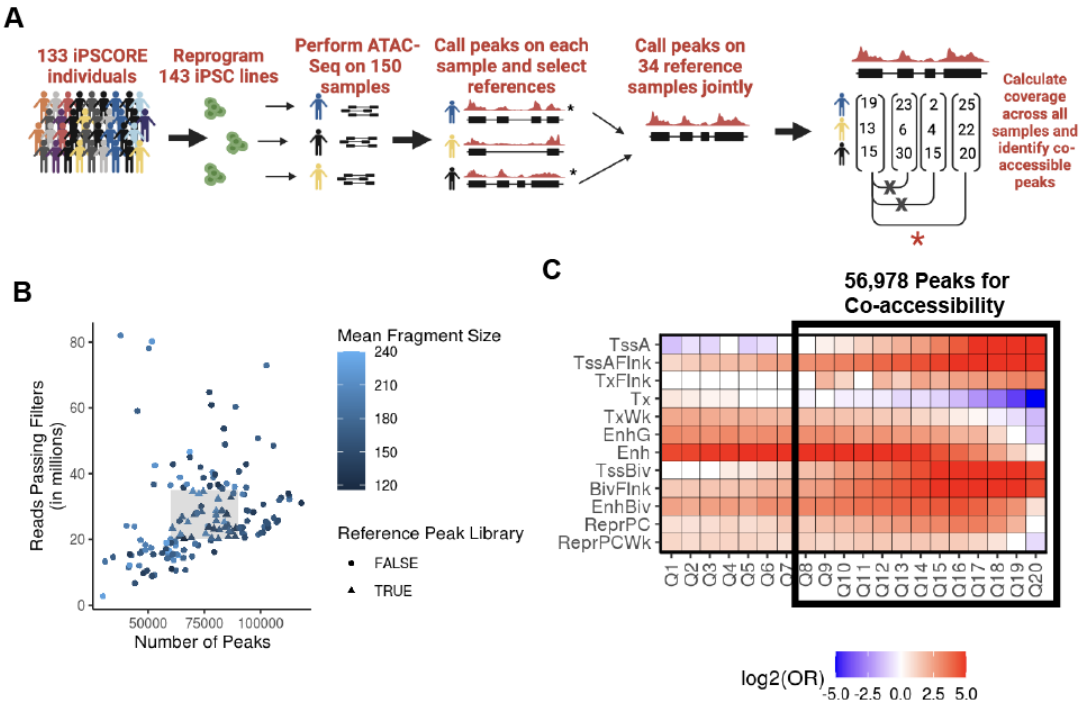

Supplemental Figure 5. ATAC-seq peak calling strategy and quality control

(A) Diagram of ATAC-seq peak calling strategy. We set out to identify a reference set of high quality ATAC-seq peaks to use the co-accessibility analysis (see Methods for a detailed description). Briefly, we first used MACS2 to call broad peaks on each of the 150 ATAC-seq samples individually. We then evaluated the quality of each sample by examining the number of reads passing filters, the mean fragment size, and the number of broad peaks. Using these quality metrics, we selected 34 reference samples from unrelated iPSCORE individuals and used MACS2 to call narrow peaks on them jointly to establish a set of 136,333 reference peaks (including 132,225 autosomal peaks and 4,108 peaks on sex chromosomes, MACS2 score > 100). We then used *featureCounts* to count the number of reads in each of the 136,333 reference peaks for each of the 150 ATAC-seq samples. We selected 56,978 autosomal peaks (see details in Methods) and calculated co-accessibility. Created with BioRender.com.

(B) Scatter plot showing quality metrics of the 150 ATAC-seq samples. As described above, we evaluated the quality of samples based on three technical variables; number of reads passing filters, mean fragment size, and number of broad peaks. In the plot, each point represents an ATAC-seq sample, the box represents the window where we selected the 34 reference samples (triangles) from unrelated iPSCORE individuals.

(C) Heatmap showing the enrichment of ATAC-seq peaks in hiPSC-18 ChromHMM chromatin states by MACS2 score quantile. To use chromatin state enrichments in order to prioritize ATAC-seq peaks for downstream co-accessibility analysis, we binned the peaks into 20 quantiles by their MACS2 score and

created bed files containing the coordinates for peaks in each quantile. We then calculated their enrichment (Odds Ratio) in each of the 12 active chromatin states for hiPSC-18, using *bedtools fisher*. It has been extensively shown that ATAC-seq identifies accessible chromatin that is enriched with active regulatory elements (TssA, Enh), bivalent chromatin (TssBiv, EnhBiv), and repressed polycomb regions (ReprPC, ReprPCWk) in iPSCs, therefore we evaluated the enrichment of these chromatin states across the 20 quantiles. We noticed that TssA are enriched in quantiles 8-20 and Enh are enriched across all 20 quantiles. Additionally, TssBiv and ReprPC started to exhibit diminishing enrichment around quantile 8. These observations supported our decision to use peaks in quantiles 8-20 (annotated by the black box) for downstream analyses.

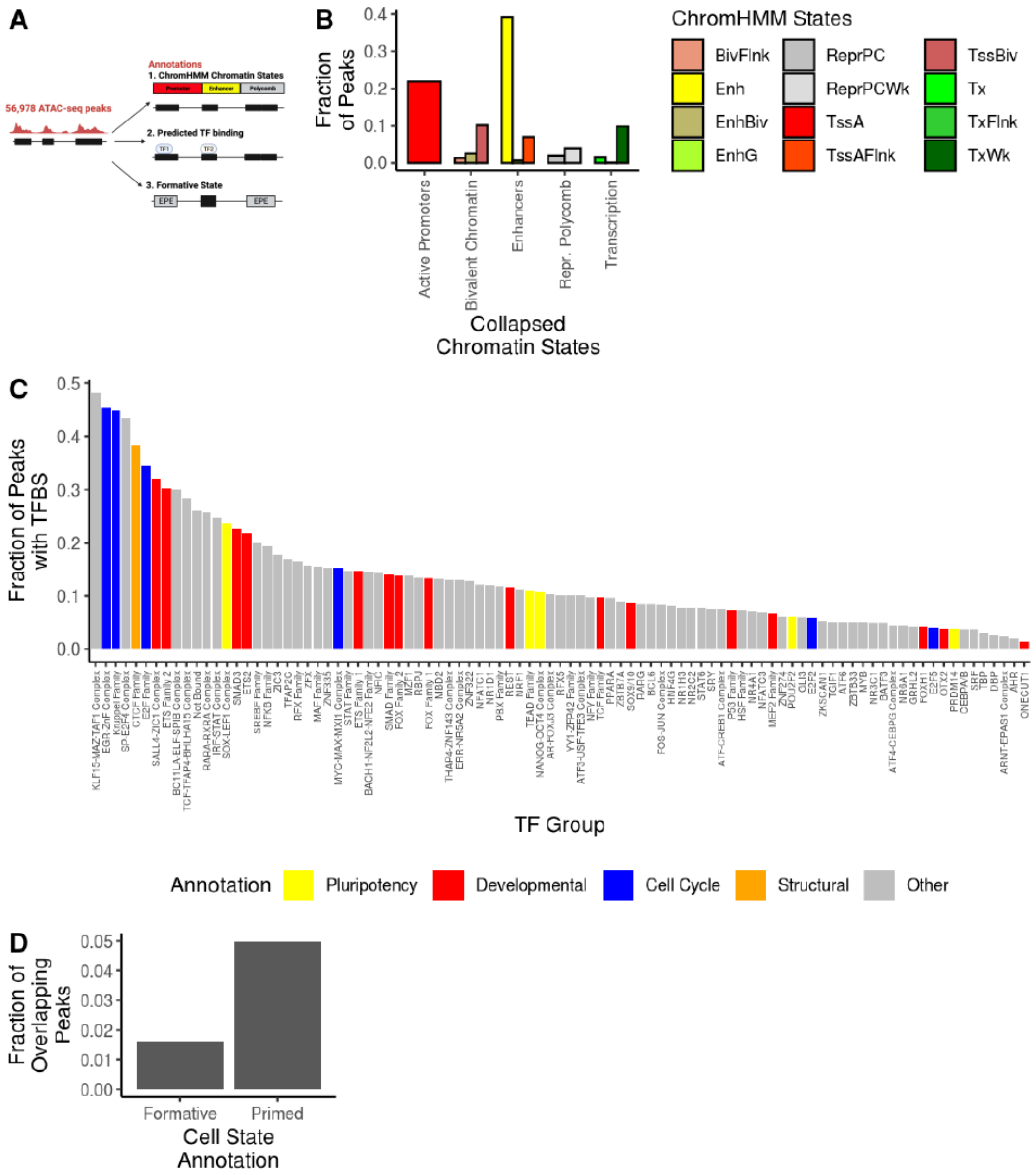

Supplemental Figure 6. Epigenetic Characterization of Reference ATAC-seq Peaks

(A) Diagram of Epigenome Annotation Strategy. The 56,978 ATAC-seq peaks were characterized with three epigenetic annotations; 1) ChromHMM iPSC-18 chromatin states, 2) TF binding predicted by TOBIAS (v0.15.1), and 3) formative-state associated peaks. Created with Biorender.com.

(B) Bar plot showing the fraction of ATAC-seq peaks annotated by the iPSC-18 ChromHMM chromatin states and the five collapsed chromatin states. We filtered ATAC-seq peaks in 3 inactive chromatin states (ZNF genes & repeats, heterochromatin, and quiescent chromatin). We binned the remaining 12 chromatin states into 5 collapsed states by molecular similarities. “Active promoters” (TssA) were not collapsed, the collapsed “Enhancer” annotation consisted of peaks in enhancers (Enh), genic enhancers (EnhG), and flanking active promoters (TssAFlnk), “Bivalent Chromatin” consisted of bivalent promoters

(TssBiv), bivalent enhancers (EnhBiv), and regions flanking bivalent chromatin (BivFlnk), "Transcription" consists of strong (Tx) and weak (TxWk) transcription, and flanking transcription (TxFlnk), and "Repressed Polycomb" consists of both polycomb states (ReprPC, ReprPCWk). We then calculated co-accessibility on ATAC-seq peaks in these 5 collapsed chromatin states.

(C) Barplot showing the fraction of ATAC-seq peaks bound by a selected subset of 93 TF groups. As indicated by the legend, bars are colored by an associated biological process curated by a review of the literature.

(D) Bar plot showing the fraction of ATAC-seq peaks overlapping formative or primed-associated peaks. Formative peaks overlapped GCTM-2^{high}CD9^{high}EPCAM^{high} specific peaks and primed peaks overlapped GCTM-2^{mid}-CD9^{mid} specific peaks obtained from reanalyzing the Lau et al. dataset[1].

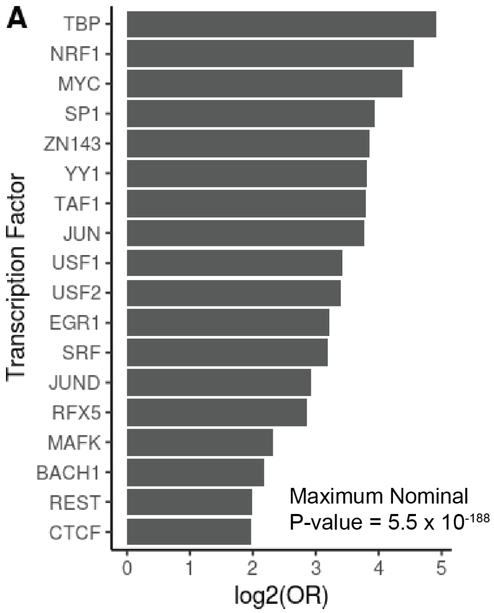

Supplemental Figure 7. Validation of TOBIAS TFBS predictions

(A) Bar plot demonstrating the accuracy of TOBIAS TFBS predictions for 18 TFs. To validate the TOBIAS TFBS binding sites, we obtained IDR-corrected TF ChIP-seq peaks from H1 embryonic stem cells for 18 transcription factors from ENCODE. We performed two-tailed Fisher's Exact tests on the predicted TFBSs for the corresponding TF based on whether there is overlap with the experimentally validated TF ChIP-seq peaks. This reveals that TOBIAS predicted TFBSs were highly enriched in experimentally validated ChIP-seq peaks (maximum p-value = $5.5x10^{-188}$, Supplemental Data 9).

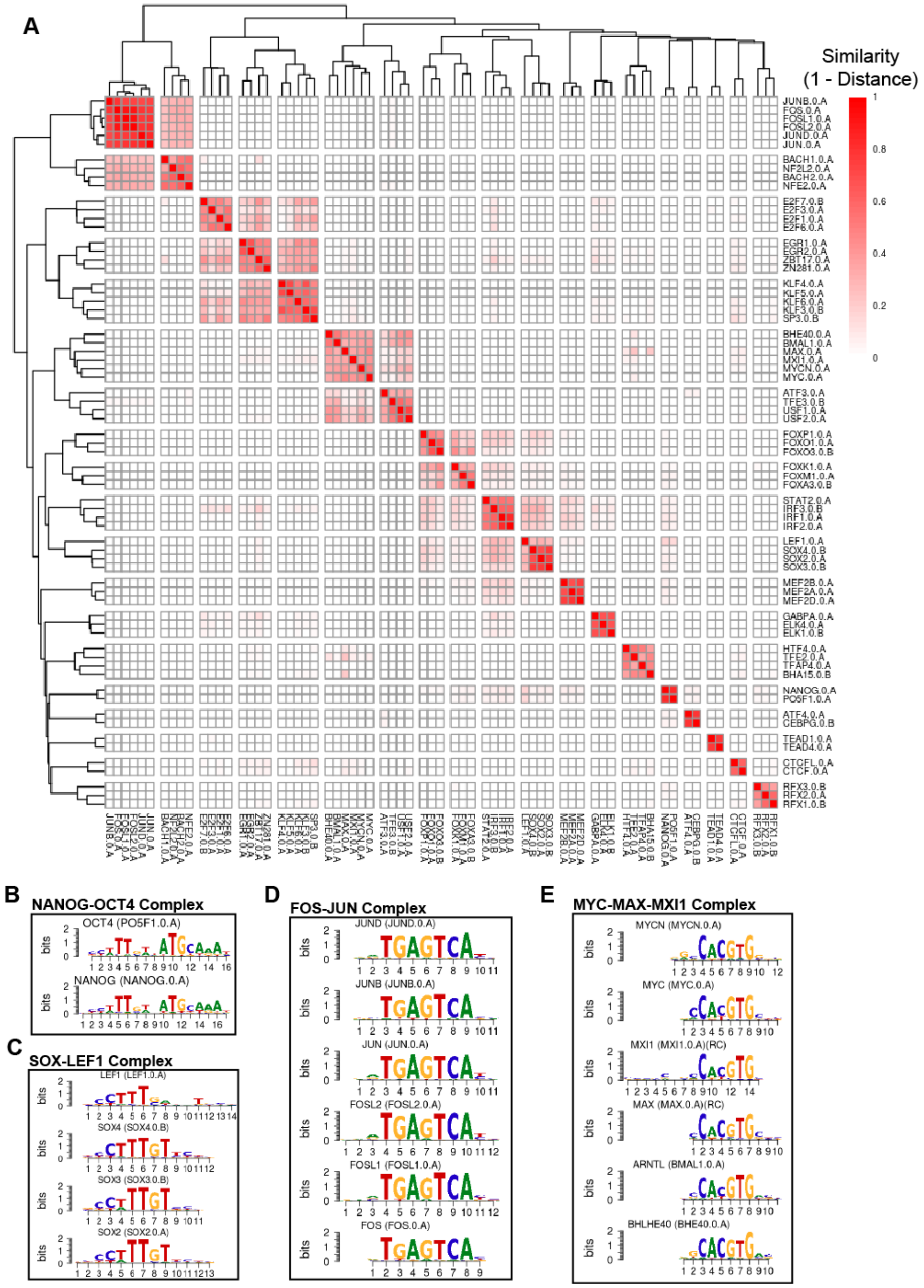

Supplemental Figure 8. Defining TF groups by binding site similarity

(A) Heatmap displaying TFBS similarities for collapsed TF groups. To account for motif similarity of closely related TFs and co-binding TFs that form complexes, we used the motif distance matrix from the TOBIAS output to binding site similarities (1 – distance) across 187 motifs. We used *cutree* (h = 0.75) to collapse the 187 motifs into 92 TF groups (Supplemental Data 10). For plot legibility, only 19 collapsed groups consisting of 68 motifs are shown.

(B-E) Consensus sequences of motifs collapsed into TF groups. (B) NANOG.0.A and PO5F1.0.A (OCT4) have similar, long motifs that capture the distinct binding sequences for both TFs and form the NANOG-OCT4 Complex. (C) SOX TFs (SOX2, SOX3, and SOX4) have similar motifs to LEF1, thus form a complex, (D) TFs from the FOS and JUN families have highly similar motifs and form the FOS-JUN Complex. (E) Others have demonstrated that MYC (MYC.0.A) and MYCN (MYCN.0.A) form complexes with MAX, MXI1, ARTNL (BMAL.0.A) and BHLHE40 (BHE40.0.A), and collectively they form the MYC-MAX-MXI1 Complex. The plot titles include the standard gene name, and the motif name.

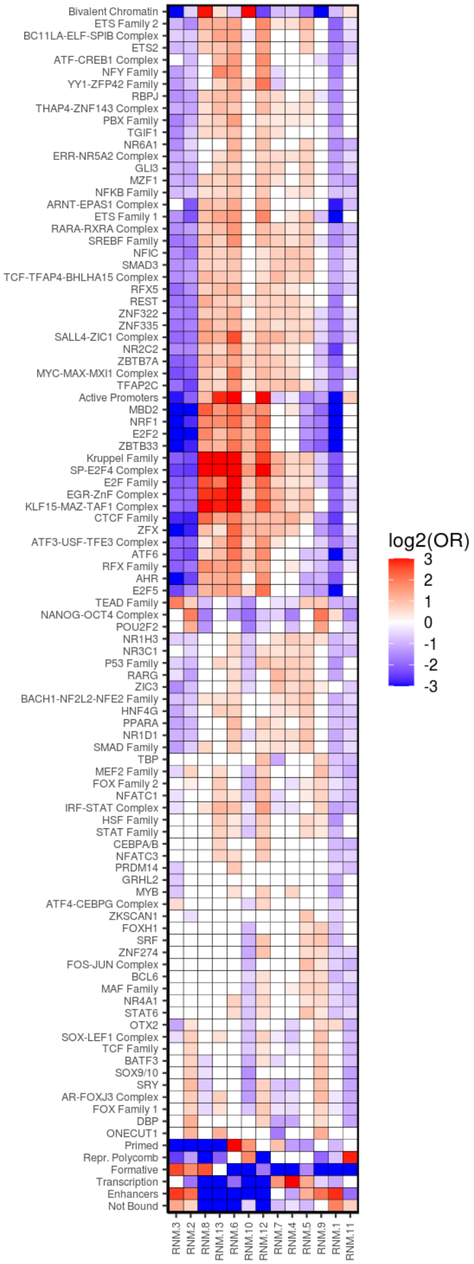

Supplemental Figure 9. RNM Enrichments with Three Classes of Epigenetic Markers

Heatmap displaying epigenetic annotations of the 13 major RNMs. This figure is a comprehensive version of Figures 3A-C and consists of RNM enrichments of 1) all 92 TF groups and "Not Bound" peaks; 2) 5 collapsed hiPSC chromatin states (Supplemental Figure 6B), and 3) two self-renewal cell states (Formative-state and primed-state associated peaks (Supplemental Figure 6D). Only significant enrichments are shown (non-significant enrichments are filled in white). The results of this analysis are reported in Supplemental Data 12.
