# Supplementary material for: Complex regulatory networks influence pluripotent cell state transitions in human iPSCs
Source: Nat Commun. 2024 Feb 23;15:1664. doi: 10.1038/s41467-024-45506-6 (PMC10891157; doi:10.1038/s41467-024-45506-6)
Supplement: Supplementary file 3 — Description of Additional Supplementary Files [file 41467_2024_45506_MOESM3_ESM.pdf]

Description of Additional Supplementary Files

File Name: Supplemental Data 1
Description: iPSCORE Subject Information. Information about each of the 219 iPSCORE individuals that were included in this study, including subject iPSCORE ID (Column A), subject universally unique identifier (UUID, Column B), whole genome sequencing sample UUID (Column C), sex (Column D), age at enrollment (Column E), if there is corresponding RNA-seq data (Column F), ATAC-seq data (Column G), and the top 20 genotype PCs for global ancestry (Columns H-AA) for the individual. The kinship matrix describing the relatedness of the samples was deposited on Figshare.

File Name: Supplemental Data 2
Description: RNA-seq Sample Information. Information about the 213 RNA-seq samples used in this study, including subject iPSCORE ID (Column A), subject universally unique identifier (UUID, Column B), RNA-seq sample UUID (Column C), the iPSC iPSCORE ID which consists of the iPSCORE ID, iPSC clone number and the iPSC passage number (Column D), the iPSC clone (Column E) and passage (Column F) numbers, the number of properly paired reads (Column G), the estimated formative proportion (Column H), and the CIBERSORT deconvolution correlation values (Column I).

File Name: Supplemental Data 3
Description: Signature Gene Matrix for RNA-seq Cellular Deconvolution. Signature gene expression matrix for CIBERSORT cellular deconvolution of 213 RNA-seq samples. The table includes the Gencode v34 gene ID and gene name (Columns A and B), the expression (TPM) of 300 differentially expressed genes between the FACS-sorted formative (GCTM-2^{high}CD9^{high}EPCAM^{high}, Column C) and primed (unsorted, column D) populations from Lau et al. 2020[1]

File Name: Supplemental Data 4
Description: Gene Network Module Memberships. Information about gene network module (GNM) analysis annotations for the 16,110 expressed genes, including the Gencode v34 gene ID (Column A) and gene name (Column B), the corresponding GNM (Column C), whether the corresponding GNM was used for downstream analyses (Major GNM, Column D), whether the gene was considered Pareto (Column E), the gene's co-expression degree connectivity across all 16,110 expressed genes (genome-wide degree, Column F), and the gene's co-expression degree connectivity (Column G) within its corresponding module (intramodular degree).

File Name: Supplemental Data 5
Description: GNM Enrichment Results. Results from the Fisher's Exact test to calculate pluripotency cell state gene set enrichments. Information includes the annotation tested

(Column A), the GNM tested (Column B), the odds ratio (Column C), the two-sided p-value (Column D), and the Benjamini-Hochberg corrected p-value (Column E).

File Name: Supplemental Data 6
Description: ATAC-seq Sample Information Information. about the 263 individual ATAC-seq samples from iPSCORE individuals that were merged by library into 150 ATAC-seq samples. The table includes the subject universally unique identifier (UUID, Column A), the iPSCORE subject ID (Column B), the iPSC iPSCORE ID which consists of the iPSCORE subject ID, clone number and passage number (Column C), sample UUIDs for the 150 merged ATAC-seq libraries (Column D), sample UUIDs for the 263 ATAC-seq samples before merging (Column E), the iPSC clone number (Column F), the iPSC passage (Column G) and number of reads passing filters (Column H), the mean fragment size (Column I), the number of broad peaks used for QC (Column J), the ratio of 100bp reads to 150bp reads in the merged sample (1=100% 100bp reads and 0=100% 150bp reads; see Methods; Column K), the estimated formative proportion (Column L), the CIBERSORT deconvolution correlation values (Column M), and whether the merged sample was used as a reference for establishing a set of reference narrow peaks (Column N). Note: To obtain non-redundant data for the 150 merged ATAC-seq samples used in downstream analyses, use the unique rows from columns A-D and F-M.

File Name: Supplemental Data 7
Description: Signature Peak Matrix for ATAC-seq Cellular Deconvolution. Signature gene expression matrix for CIBERSORT cellular deconvolution of 150 ATAC-seq samples. The table includes the narrow peak ID (Column A), the accessibility (TMM) of 200 differentially accessible peaks between the FACS-sorted formative (GCTM-2^{high}CD9^{high}EPCAM^{high}, Column B) and primed (GCTM-2^{mid}-CD9^{mid}, column C) populations from Lau et al. 2020.

File Name: Supplemental Data 8
Description: Regulatory Network Module (RNM) Memberships and Annotations. Information about regulatory network module (RNM) analysis annotations for the 56,978 accessible peaks, including the peak ID (Column A), peak chromosome, start and end positions (Column B-D), the corresponding RNM (Column E), whether the RNM was one of the 13 used for downstream analyses (Major RNM, Column F), whether the peak was considered a Pareto peak (Column G), the peak's coaccessibility degree connectivity (Column H) across all 56,978 accessible peaks (genome-wide degree), and the peak's co-accessibility degree connectivity (Column I) within its corresponding module (intramodular degree), the iPSC-18 ChromHMM chromatin state annotation (Column J), the collapsed chromatin states (Column K), and the Gencode v34 gene ID (Column L), gene name (Column M), the distance in base pairs (Column N) of the closest expressed gene after ROCK kinase inhibitor stimulation (see Methods), and whether the peak overlaps a Formative (Column O) or Primed (Column P) peak.

File Name: Supplemental Data 9

Description: TOBIAS Predicted Binding Site Validation. This table includes information about the TOBIAS prediction validation analysis, including the ENCODE ID of the TF ChIP-seq data for H1 ESCs (Column A), the corresponding transcription factor (Column B), the two-sided p-value and the odds ratio for the Fisher's Exact tests (Columns C-D).

File Name: Supplemental Data 10
Description: Transcription Factor Group Motif Memberships. Information about TF groups determined by TOBIAS predicted binding similarities for 187 motifs, including the HOCOMOCO motif ID (Column A), the Gencode v34 gene ID (Column B), gene name (Column C) and the name of the collapsed TF group to which the motif belongs (Column D). Note: The TOBIAS motif distance matrix and predicted binding sites for all 187 motifs were uploaded to GEO (GSE203377) and FigShare (136585).

File Name: Supplemental Data 11
Description: Annotations of 56,978 peaks for binding of 92 TF groups. This table includes the TOBIAS-predicted transcription factor binding sites for all 56,978 ATAC-seq peaks. Information includes; the peak ID (Column A), and binary annotations for the 92 collapsed TF groups and "Not Bound" peaks (Columns B-CP), where 1 indicates that there is a bound TF group on the corresponding peak.

File Name: Supplemental Data 12
Description: RNM Annotation Enrichment Results. This table contains the Fisher's Exact test results for enrichments in the RNM Pareto peaks, including the annotation type (transcription factor, chromatin state, and cell state, Column A), the corresponding annotation (Column B), the tested RNM (Column C), the odds ratio, two-tailed p-value, and BenjaminiHochberg corrected p-value (Columns D-F).

File Name: Supplemental Data 13
Description: RNM Fetal Tissue ATAC-seq Enrichment Results. This table contains the Fisher's Exact test results for the single-cell fetal tissue-specific peak RNM enrichments, including the tested fetal cell type (Column A), the tested RNM (Column B), the odds ratio, two-tailed p-value, and Benjamini-Hochberg corrected p-value (Columns C-E)

File Name: Supplemental Data 14
Description: Fetal Tissue TFBS Enrichment Results. This table contains the Fisher's Exact test results for the single-cell fetal tissue-specific peak TFBS enrichments, including the tested fetal cell type (Column A), the tested TFBS (Column B), the odds ratio, two-tailed p-value, and Benjamini-Hochberg corrected p-value (Columns C-E).

File Name: Supplemental Data 15

Description: Allele-Specific Chromatin Accessibility (ASCA) Results. This table contains information on SNPs tested for ASCA, including SNP ID and gnomad RSID (Columns A-B), Peak ID (Column C), number of reads mapping to the reference and alternative alleles (Columns DE), the allelic imbalance fraction (Column F), the number of heterozygous individuals tested (Column G), the minor allele frequency (Column H), the two-sided p-value from the binomial test and BenjaminiHochberg corrected p-value (Columns I-J), whether the SNP has ASCA (adjusted P-value < 0.05, Column K).
