# Supplementary material for: Complex regulatory networks influence pluripotent cell state transitions in human iPSCs
Source: Nat Commun. 2024 Feb 23;15:1664. doi: 10.1038/s41467-024-45506-6 (PMC10891157; doi:10.1038/s41467-024-45506-6)
Supplement: Supplementary file 19 — Reporting Summary [file 41467_2024_45506_MOESM19_ESM.pdf]

nature portfolio

Corresponding author(s): Kelly A. Frazer

Last updated by author(s): 2024/01/12

Reporting Summary

Nature Portfolio wishes to improve the reproducibility of the work that we publish. This form provides structure for consistency and transparency in reporting. For further information on Nature Portfolio policies, see our Editorial Policies and the Editorial Policy Checklist.

Statistics

For all statistical analyses, confirm that the following items are present in the figure legend, table legend, main text, or Methods section.

n/a	Confirmed	
☐	☒	The exact sample size (*n*) for each experimental group/condition, given as a discrete number and unit of measurement
☐	☒	A statement on whether measurements were taken from distinct samples or whether the same sample was measured repeatedly
☐	☒	The statistical test(s) used AND whether they are one- or two-sided<br>*Only common tests should be described solely by name; describe more complex techniques in the Methods section.*
☐	☒	A description of all covariates tested
☐	☒	A description of any assumptions or corrections, such as tests of normality and adjustment for multiple comparisons
☐	☒	A full description of the statistical parameters including central tendency (e.g. means) or other basic estimates (e.g. regression coefficient) AND variation (e.g. standard deviation) or associated estimates of uncertainty (e.g. confidence intervals)
☐	☒	For null hypothesis testing, the test statistic (e.g. *F*, *t*, *r*) with confidence intervals, effect sizes, degrees of freedom and *P* value noted<br>*Give P values as exact values whenever suitable.*
☒	☐	For Bayesian analysis, information on the choice of priors and Markov chain Monte Carlo settings
☐	☒	For hierarchical and complex designs, identification of the appropriate level for tests and full reporting of outcomes
☐	☒	Estimates of effect sizes (e.g. Cohen's *d*, Pearson's *r*), indicating how they were calculated

Our web collection on statistics for biologists contains articles on many of the points above.

Software and code

Policy information about availability of computer code

Data collection	Chromatin state data were obtained from the Roadmap Epigenomics repository, transcription factor motif position weight matrices were obtained from HOCOMOCO version 11, and the fetal ATAC-seq data were obtained from the Descartes Atlas of Human Chromatin Accessibility during Development. All data was downloaded using the wget command.
Data analysis	Data analysis was performed using R (version 4.0.1) and several commercially-available packages listed below;<br>- coxme (version 2.2-17)<br>- igraph (version 1.3.2)<br>- edgeR (version 3.30.3)<br>- matrixStats (version 0.52.2)<br>- preprocessCore (version 1.51.1)<br><br>WASP (version 0.2.2) and ASEReadCounter (version 3.4-46) were used to calculate allele-specific chromatin accessibility.<br><br>All scripts developed to perform this study are available in GitHub (https://github.com/tdarthur40/ipsc_coaccessibility) and citable through Zenodo (https://zenodo.org/records/10481265).

For manuscripts utilizing custom algorithms or software that are central to the research but not yet described in published literature, software must be made available to editors and reviewers. We strongly encourage code deposition in a community repository (e.g. GitHub). See the Nature Portfolio guidelines for submitting code & software for further information.

Data

Policy information about availability of data

All manuscripts must include a data availability statement. This statement should provide the following information, where applicable:

- Accession codes, unique identifiers, or web links for publicly available datasets
- A description of any restrictions on data availability
- For clinical datasets or third party data, please ensure that the statement adheres to our policy

The 222 iPSCORE hiPSC lines are available through WiCell Research Institute (www.wicell.org; NHLBI Next Gen Collection). The previously published FASTQ sequencing data for bulk RNA-seq and WGS have been deposited in dbGaP under the accession codes phs000924 (https://www.ncbi.nlm.nih.gov/projects/gap/cgi-bin/study.cgi?study_id=phs000924.v4.p1) and phs001325 (https://www.ncbi.nlm.nih.gov/projects/gap/cgi-bin/study.cgi?study_id=phs001325.v5.p1), respectively. The bulk ATAC-seq FASTQ files generated in this study have been deposited in Gene Expression Omnibus (GEO) under the accession code GSE203377 (https://www.ncbi.nlm.nih.gov/geo/query/acc.cgi?acc=GSE203377). The TOBIAS transcription factor binding predictions, the TMM-normalized counts, the kinship matrix, and the reference narrow peak file have been deposited on Figshare (https://figshare.com/account/home#/projects/136585). Source data are provided with this paper.

Research involving human participants, their data, or biological material

Policy information about studies with human participants or human data. See also policy information about sex, gender (identity/presentation), and sexual orientation and race, ethnicity and racism.

Reporting on sex and gender	There were data from 219 subjects from the iPSCORE collection, including 97 males and 122 females used in this study. Sex was controlled for by including it as a covariate in the linear mixed model. Multiple ethnicities were represented in the data. Ethnicities were not directly accounted for, instead they were indirectly accounted for by using the top 20 PCs for global ancestry as covariates in the linear mixed model.
Reporting on race, ethnicity, or other socially relevant groupings	As highlighted above, this study includes data generated from ethnically-diverse iPSCORE subjects. Subjects were not partitioned based on ethnicity, instead global ancestry was included as a covariate to correct for biological variability. The goal of the study is to characterize the epigenome which is mainly influenced by non-genetic factors, therefore we corrected for genetic factors.
Population characteristics	Our group previously published the PCs for global ancestry for all iPSCORE subjects. We used the top 20 PCs for global ancestry in this study.
Recruitment	The recruitment for the iPSCORE collection has been extensively detailed in previous publications from the consortium.
Ethics oversight	Recruitment of these individuals was approved by the Institutional Review Boards of the University of California, San Diego, and The Salk Institute (project no. 110776ZF).

Note that full information on the approval of the study protocol must also be provided in the manuscript.

Field-specific reporting

Please select the one below that is the best fit for your research. If you are not sure, read the appropriate sections before making your selection.

[x] Life sciences   [ ] Behavioural & social sciences   [ ] Ecological, evolutionary & environmental sciences

For a reference copy of the document with all sections, see nature.com/documents/nr-reporting-summary-flat.pdf

Life sciences study design

All studies must disclose on these points even when the disclosure is negative.

Sample size	The sample size was dictated by the number of samples that we successfully generated molecular data for. We previously generated RNA-seq data for iPSCs derived 222 iPSCORE subjects of which 213 had paired whole genome sequencing. For this study, we successfully generated 150 ATAC-seq libraries for 143 iPSC lines derived from 133 iPSCORE subjects. Six subjects only had ATAC-seq, resulting in a total of 219 iPSCORE subjects with data used in this study.
Data exclusions	In the manuscript, we highlight quality control thresholds that were used to determine which ATAC-seq samples would be included in the analysis. Libraries that did not meet the quality thresholds for number of reads passing filters, number of peaks and fragment size were discarded.
Replication	In the study, we systematically generated 150 ATAC-seq libraries for 143 iPSC lines from 133 iPSCORE subjects. Seven iPSC lines had multiple libraries which served as technical replicates. Additionally, we extensively outlined the procedure for sample processing and analysis in the Methods to ensure that the data can be reliably reproduced.
Randomization	Samples were not grouped, therefore the study design was not suitable for randomization. We accounted for variability by the inclusion of covariates for technical (ie. number of reads, passage number, etc.) and biological (ie. sex, global ancestry, etc.) attributes in the linear mixed model.

Blinding	Blinding was not applicable to the study design because the technical and biological variability was controlled for using covariates. Sample classification was not required to achieve the objective of the study.

Reporting for specific materials, systems and methods

We require information from authors about some types of materials, experimental systems and methods used in many studies. Here, indicate whether each material, system or method listed is relevant to your study. If you are not sure if a list item applies to your research, read the appropriate section before selecting a response.

Materials & experimental systems

n/a	Involved in the study	
☒	☐	Antibodies
☐	☒	Eukaryotic cell lines
☒	☐	Palaeontology and archaeology
☒	☐	Animals and other organisms
☒	☐	Clinical data
☒	☐	Dual use research of concern
☒	☐	Plants

Methods

n/a	Involved in the study	
☒	☐	ChIP-seq
☒	☐	Flow cytometry
☒	☐	MRI-based neuroimaging

Eukaryotic cell lines

Policy information about cell lines and Sex and Gender in Research

Cell line source(s)	iPSCs were derived from 219 iPSCORE subjects. The derivation protocol was extensively detailed in previous publications from the iPSCORE consortium.
Authentication	The derivation was conducted internally in Dr. Kelly A Frazer's lab. To confirm sample labeling, sample identity check was performed on all iPSCs using their genotype data.
Mycoplasma contamination	All lines were routinely checked for mycoplasma contamination and tested negative.
Commonly misidentified lines (See ICLAC register)	This doesn't apply to the study because the iPSCs were derived internally and their identities were confirmed.
